# Student support in higher education: campus service utilization, impact, and challenges

**DOI:** 10.1016/j.heliyon.2022.e12559

**Published:** 2022-12-22

**Authors:** Chithira Johnson, Rizwan Gitay, Abdel-Salam G. Abdel-Salam, Ahmed BenSaid, Radwa Ismail, Rusol Adil Naji Al-Tameemi, Michael H. Romanowski, Batoul Mohamad Kazem Al Fakih, Khalifa Al Hazaa

**Affiliations:** a Advisory, Student Experience Department, Qatar University, Doha, Qatar; b Senior Retention Specialist, Academic Advising Cente, Qatar University, Doha, Qatar; c Associate Professor of Statistics and Head of Student Data Management-Vice President for Student Experience Department, Student Affairs Sector, VPSA Office, Qatar University, Doha, Qatar; d Senior Data Analyst, Student Data Management-Student Experience Department, Student Affairs Sector, Qatar University, Doha, Qatar; e Master Student of Applied Statistics and Graduate Assistant –Student Experience Department, Qatar University, Doha, Qatar; f Student Experience Department, Qatar University, Doha, Qatar; g Professor Educational Research Center College of Education Qatar University, Doha, Qatar; h Graduate Assistant, Student Experience Department, Qatar University, Doha, Qatar; i Director of Student Experience Department, Vice President for Student Affairs Office, Qatar University, Doha, Qatar

**Keywords:** Student support, Campus services, At-risk students, STEM

## Abstract

Worldwide, university student support services facilitate student performance, contribute to students' success, and increase students' chances of degree completion. Student support services programs' success depends on students' help-seeking behavior. This study explores the help-seeking behavior of Foundation Program and Undergraduate students at Qatar University (QU) through their use of campus services to better understand students' use of these services. The study examines the association between help-seeking behavior, as indicated through services, on student success and persistence in two consecutive semesters, Spring 2019 and Fall 2019. Findings report a significant association between students' services and student success and persistence. A significant difference was reported between at-risk students’ majors and at-risk students in STEM and non-STEM majors. Also, there was a difference in the help-seeking behavior among males and females, nationals and non-nationals, and student classifications.

## 1 Introduction

Worldwide, campus support services are available to students in educational institutes, national and international, to facilitate student performance (Perez-Encinas and Ammigan, 2016), and contribute to students' success in their academic and career plans (Lenz-Rashid, 2018). Similarly, Okpych et al. (2020) indicated that support services were established to increase the chances of students continuing in college and meeting their academic needs, ultimately leading to completing their studies and enriching the skills necessary for college success.

While institutions are extensively working on students' positive experiences and launching several student support services programs, the success of these programs depends mainly on the student's help-seeking behavior (Okpych et al., 2020). Students need to know and understand what they are learning to avoid trouble, with which material to study, for how long, and when to seek help. Seeking help is associated with higher academic ability and positive teacher-student relationships (Chu et al., 2018). From this perspective, it can be stated that students' help-seeking behavior is a substantial campus support service outcome. Saleh et al. (2017) have reported that about 90% of students experience problems in their academic lives, such as stress, future planning, anxiety, and depression. In contrast, only 15% of students reported seeking help from different sources. A possible reason for this low response can be attributed to a reluctance to seek help (Dyrbye et al., 2015).

Similarly, Eisenberg et al. (2007) commented that lack of resources, cultural differences, social stigma, and hesitations are significant factors that hinder students from seeking help. In this matter, Finney et al. (2018) mentioned that educators need to explain the perceived benefits, such as learning, and costs, such as being labeled as scientifically backward, that come between the relationship between help-seeking behavior and achieving students' desire for success. According to Roussel et al. (2011), the perceived benefits reflect the understanding that seeking help is a valuable strategy that promotes learning. On the contrary, the estimated costs reflect the threat to students' self-esteem caused by recognizing their need for assistance.

In Qatar, obtaining a higher education is integral to success, employment, and future opportunities (Khan and Awan, 2017). Education is prominent in Qatar's National Vision 2030 and National Development Strategy and is essential to the transition to a knowledge economy. This is just one indicator of the national importance of the catalytic role of education. Since education could be a substantial predictor of innovation and economic productivity at times, higher educational institutions should support their students and provide campus services. According to Chao et al. (2018), having a learning environment that assists students in identifying their problems and seeking help from on-campus support services enhances their motivation to study and promotes their academic performance.

There is a broad spectrum of services offered to students. For example, the university provides the following student services Academic Advising and Guidance (services to aid in students meeting their academic goals); Career Counseling and Exploration (counseling and professional development services to prepare students to compete for the best career opportunities); Student Counseling (services to students to overcome psychological, behavioral, social and emotional disorders and difficulties that may affect their performance); Writing and Language Support, Academic Support (i.e., peer tutoring, study skills workshops); and Inclusion and Accessibility support (See Qatar University Students Affairs, 2022 for additional information regarding student services at Qatar University). Most research on student services seems to focus on a single campus service and any association between usage, persistence, and retention. It is essential to understand how the use of on-campus services may influence students’ persistence and retention. However, from what we have experienced, this research area has not yet been sufficiently explored.

With that in mind, the purpose of this study is to address if students and students considered at-risk seek help by using any support services available on campus. More specifically, the research aims to explore if any associations exist between the usage of student services and these students’ success and persistence and if there are any differences in the help-seeking behavior as indicated by gender, nationality, major, and student classification.

Therefore, this study investigates the use, impact, and challenges of student support services in higher education. More specifically, the study aims to answer the following questions:

1a. To what extent do students seek help using the support services available on campus?
1b. To what extent do students identified as at-risk seek help by using the support services available on campus?
2a. Is there an association between the usage of campus services by all students and their success?
2b. Is there an association between the usage of campus services by students identified as at-risk and their success?
3a. Is there an association between the usage of campus services by all students and their persistence?
3b. Is there an association between the usage of campus services by students identified as at-risk and their persistence?
4a. Is there a difference in the help-seeking behavior of students in general, as indicated through the usage of campus services based on some students' demographic variables, namely, gender, nationality, major, and student classification?
4b. Is there a difference in the help-seeking behavior of students identified as at-risk, as indicated through the usage of campus services based on some students' demographic variables?

### 1.1 Context

The current research has been motivated by the gaps in our knowledge of existing patterns of service usage by students, lack of understanding around the help-seeking behavior of differing student cohorts, knowledge of the demographic factors that could influence help-seeking behavior, desire to ascertain the offering of right services to the right students in a timely and efficient manner and the need to validate the link between service utilization and student success.

### 1.2 Literature review

This section presents a brief but focused review of literature about high school students’ help-seeking behavior, how students can benefit by using support services on campus, and factors that impact support services' usage.

#### 1.2.1 Students’ help-seeking behavior

According to Ochi et al. (2018), help-seeking behavior is a person’s behavior toward pursuing and asking for help from others by speaking up about one’s problem. Disabato et al. (2018) defined help-seeking as acquiring help to resolve specific issues inside and outside the campus. Students' help-seeking behaviors refer to students requiring assistance in different matters, such as academic problems and stress. Saleh et al. (2017) found that very few students express their problems for support.

In addition, students show very reluctant help-seeking behavior, as they feel hesitant to ask for help. Disabato et al. (2018) postulated that the most significant barriers to college students’ help-seeking were trust in mental health professionals, embarrassment, poor mental health literacy, and an inclination to self-reliance. For instance, Dyrbye et al. (2015) found that 33.9% of students in several universities in the United States suffering from burnout come to ask for help from student services.

Research has demonstrated that most college students face challenges in seeking help with academic issues (Chao et al., 2018; Karabenick and Knapp, 1988). In these cases, Newman (2000) points out that these difficulties can become barriers to obtaining the resources needed to solve the problem, often making modest efforts to resolve academic problems, stopping early, or becoming content with being unsuccessful.

Bornschlegl et al. (2020) report that since all higher educational institutions have different social setups and cultural norms, students’ attitudes toward help-seeking and the facilities available to gain this help vary immensely. Hence, educational institutes should tailor the academic support facilities to the attitudes and needs of their student base.

Seeking help is governed by several factors, both academic and non-academic (Karabenick and Newman, 2013; Lotkowski et al., 2004). Students who seek help for their problems undergo improved adjustment experiences and face fewer emotional and behavioral adaptations (Fallon and Bowles, 2001; Watson, 2005). This clarifies that the attitude held by an individual towards help-seeking behavior is the driver of the process of change (McCarthy and Holliday, 2004). Therefore, students’ attitudes toward help-seeking are reliable indicators of the likeliness of seeking help available in their institutions if they face academic or non-academic issues.

#### 1.2.2 How students benefit by using support services on campus

Research reports a positive association between students' usage of specific on-campus support services and their persistence within the academic institution. Student retention and sustainability can be affected by the ability to pay for college (Butler, 2011; O'Keeffe, 2013). According to Duniway (2012), a university’s financial aid is critical in ensuring that students continue and complete their degrees. The results indicated that students obtaining higher grants and scholarships were more likely to remain at and graduate from their first college. Ganem and Manasse (2011) report that scholarships are one of the strongest predictors of student persistence, progression, and timely graduation. Robbins et al. (2004) financial support played a statistically significant role in predicting college continuity.

It can be argued that on-campus student support services are the best option for students who feel uncomfortable discussing their issues at home. In addition to supporting the students emotionally, university services assist them with their academic problems, such as tutoring and recommending remedial courses (Bettinger et al., 2013; Laskey and Hetzel, 2011). Chao et al. (2018) argue that students can achieve self-regulation and learning outcomes while seeking help to learn independent problem-solving and self-determination. Counselors in higher educational institutions provide many benefits to students, such as helping them prepare for academics and social challenges (Abiola and Paul, 2019). Counselors can also motivate students and facilitate the process of career planning. Moreover, they can encourage students to discuss their problems openly with their parents or guardians (Jackson, 2017).

Kuh et al. (2010) argue that students should have access to learner-centered support services to increase student persistence, such as peer tutoring and dedicated labs for writing and mathematics. Most academic services provide tutoring centers that offer academic support in speaking and writing (Roberts and Styron, 2009). Research has demonstrated that academic services significantly impact student persistence (Adelman, 1999; Pascarella and Terenzini, 2005). For example, Stewart et al. (2015) report that “support services such as tutoring, mentoring, counseling services, early intervention systems, and financial aid assistance will improve study participants’ academic deficiencies and increase persistence beyond the first year” (p. 12).

#### 1.2.3 Factors that impact the usage of support services by students

Several factors affect a student’s help-seeking behavior, such as gender (Magaard et al., 2017), ethnicity or nationality (Disabato et al., 2018), academic performance (Rafal et al., 2018), and student classification (Clark, 2005).

### 1.3 Gender

Previous studies have reported that gender significantly influences individuals' help-seeking behavior and academic self-efficacy (Baji, 2019; Drago et al., 2018). A number of studies have found that females possess more positive help-seeking attitudes than males and are more likely to recognize and accept needed services success (Aoun et al., 2004; Bergeron et al., 2005; Xie and Xie, 2019). Therefore, they are more likely to exhibit help-seeking behavior when facing issues that can hamper their academic success. On the other hand, male students are doubtful to seek help from a professional or even from their social circle in case of any distress (Sheu and Sedlacek, 2004). Xie and Xie (2019) found that gender roles moderated the association between academic help-seeking behavior and self-efficacy. They identified that females were less likely to adopt a maladaptive academic help-seeking behavior once they encountered academic difficulties.

### 1.4 Nationality

Disabato et al. (2018) discussed that in addition to gender, ethnicity, or nationality of the individual facing an issue or a mental health problem also impacts their help-seeking behaviors. Ethnic minorities in any region show less readiness to ask for help. In a US-based study, Asians and African Americans showed low rates of seeking professional help. They also had a negative attitude toward seeking help compared to European-Americans (Gee et al., 2020; Wong et al., 2014). Researchers found that while ethnic groups do not show the likelihood of seeking help from professionals, they do seek help from informal sources, like religious leaders (Brown et al., 2020; Cauce et al., 2002). However, this is regulated based on knowing a person who has used these resources. If seeking help is considered acceptable by society, individuals are more willing to seek help (Disabato et al., 2018).

Kilinc and Granello (2003) indicated that US minority students favored seeking help from peers (50%) instead of traditional sources, such as psychologists (14%), counselors (11%), psychiatrists (8%), and academic advisers (2%). It is worth mentioning that “not seeking help from anyone” was also an option among those students, and 12% responded in this manner. In contrast, in Japan, Nguyen et al. (2019) found no statistically significant difference between minority and non-minority students in formal help-seeking behaviors.

### 1.5 Academic performance

Many studies have listed academic performance and success as an outcome of help-seeking behavior (Rafal et al., 2018). However, in the context of this research, the authors propose that academic performance can also be a decisive factor in seeking help. If the student has previously witnessed success in their academic life but is experiencing problems and failures due to some inherent issues, they would need to seek help from a professional to understand how to resolve these issues and return to their prior academic success. However, students who have continued to experience academic problems will feel discouraged from visiting the help providers because they fear social rejection (Karabenick, 2003). In addition, Wimer and Levant (2011) reported that college students with the highest and lowest scores tend not to seek help. In contrast, students with moderate scores (that is, the 'C+' range) reported the most help-seeking behavior, indicating that some students most in need of help were the least likely to seek help.

### 1.6 Student’s class

The attitude and behavior of the students towards seeking academic help and advising facilities vary with the years they have spent in the institution. According to Clark (2005), the first and the last year are among the most critical years for advising. Junior and sophomore years highly impact overall student retention and degree completion. The transition that students undergo between high school and college can be a challenging experience for most first-year students as they may face some academic, social, and cultural challenges and may develop stress, depression, and maladaptive behaviors. These issues can lead to academic and non-academic failures. However, among other authors, Pace et al. (2018) identified that awareness about help facilities available, the stigma associated with help-seeking behavior, and peer support impact first-year students’ behavior and attitude towards help-seeking. In the second and third years, the support services focus on encouraging students to enroll in further courses to achieve degree completion (Kot, 2014; Schwebel et al., 2012). In the final year, the academic advisors encourage completing courses and advising about the students' career opportunities. The issues of stress and depression may reoccur at this stage due to the feelings of anxiety associated with the unknown expectations that the business and career world will hold for them. At this stage of their collegiate careers, students use student advising and career counseling facilities (Leao et al., 2011).

### 1.7 Major classification

Many students who complete high school lack preparedness for the expectations that college courses demand (Moore et al., 2010). The number of students that join colleges underprepared has shown to be more among the science, engineering, mathematics, and technology (STEM) streams than arts or social sciences (non-STEM) (Rodgers et al., 2014).

## 2 Research methods

Two consecutive semesters – Spring 2019 and Fall 2019, were used as the base semesters for this research. For this research, success is defined as the increase in a student's GPA at the end of the Spring 2019 and Fall 2019 semesters upon using campus services. Persistence is defined as students returning the following semester, Fall 2019 and Spring 2020. Finally, at-risk students are defined as students with a GPA of less than 2.00, especially those who were placed on probation and academic warning upon receiving a GPA below 2.00.

This research uses a data set provided by Qatar University’s institutional research department. Still, all information and forms required by Qatar University’s Institutional Review Board (QU-IRB) were submitted for the board’s review. Since the research involved collecting or studying existing data and the researchers’ inability to identify participants, QU-IRB provided an exemption (QU-IRB 1453-E/21).

### 2.1 Extraction of base data

First, the researchers extracted both semesters' active/registered students list from a standard student information system database. Only the relevant parameters of student ID, student name, gender, nationality, college, admitted term, nationality, major, GPA, and student classification were considered, thereby eliminating any other parameters irrelevant to the analysis or results of this study. As a final step in this process, the student IDs were matched against their at-risk status for the given semester, meaning students identified as at-risk per any of the pre-defined categories of academic underperformance by institutional policies.

### 2.2 Collection of data from service providers

To further collect data for analytical purposes, ten on-campus service providers were approached via email to provide the research team with the required data in line with University’s Institutional Review Board guidelines. These providers are highlighted in Table 1. The ten on-campus support providers are categorized into two types: Institutional Support Services and College Support Services. The Institutional support services cater to all undergraduate students enrolled at Qatar University. While the College support services provide support to specific courses offered by the individual colleges and for the students enrolled in these courses. Representatives from these ten service providers were provided with the same base data from Spring (2019) and Fall 2019. The service providers were requested to provide information on which students from the base data used their services and the frequency of usage in a given semester.

**Table 1:** Service Providers and their objectives.

Service Provider	Objective
Institutional Support Service	
Student Learning Support Center (SLSC)	Provides students assistance with academic coursework, writing assignments, transitioning to college life, and other services, such as tutoring sessions, supplemental instruction, and writing support, in addition to academic coaching and workshops.
Career Development Center (CDC)	Provides students with counseling, training and professional development services and helps to prepare students to engage and compete for the best career opportunities. It specializes in providing students with student employment during their study at QU, in addition to assisting them with sponsorship, internship and full-time job opportunities. The CDC also provides numerous career-related resources, programs and activities.
Academic Advising Center (AAC)	Focuses on supporting undergraduate and special students, such as academically underperforming students, achieve academic success. It offers advising sessions for students who experience academic difficulty, need assistance understanding their degree audit, want to ensure they are fulfilling their degree program’s academic requirements, or have been placed under one of the many categories of academic underperformance.
College Success Oasis	
College of Arts and Sciences (CAS)	The success oases within different colleges at this institution assist new or struggling students in paving their path to academic and long-life success. By offering academic assistance in introductory and advance courses, among those required from all students as part of the core curriculum. The success oases guide students to a strong start in their academic journey toward their chosen major. These oases also provide peer-tutoring in different areas of study, assist with course-specific drop-in tutoring through individual or group learning and review sessions, and provide pre-scheduled course-specific tutorials and workshops.
College of Business and Economics (CBE)	
College of Engineering (CENG)	
College of Sharia and Islamic Studies (CSIS)	
College of Education (CEDU)	
College of Law (LAWC)	
Foundation Program (FN)	

The *frequency of visits* was further consolidated into three actions: none, once, and twice or more. In addition, students were further classified as STEM and non-STEM based on students' major and concentration with the aid of Turner and Brass's (2014) report (Appendix A). Furthermore, the Success Oases, in particular, were requested to provide the list of courses for which they offered tutoring services or supplemental instruction in each of the two semesters. Only students registered in these courses were considered the sample for the success oases services.

### 2.3 Sample

The total number of active/registered students as an *overall sample* from Spring (2019) and Fall 2019 were 17,137 and 18,393, respectively. However, given the analytical requirements of this study, only students who had registered for courses belonging to the tutoring offered by the Success Oasis of a specific college were considered to generate the results of the success oasis particular to that college. However, for the other three centers being institutional services, i.e., the AAC, the CDC, and the SLSC, the data sets were analyzed in light of the frequency of utilizing the services throughout the semester as per the following parameters only: gender, at-risk status, and nationality, for the entire student population registered in the institution in a given semester.

### 2.4 Statistical analysis

Statistical analyses were performed using IBM SPSS Statistics. Since our objective is to define the characteristics of students who use the support services at Qatar University, simple yet effective statistical methods have been used to compare groups of students with different traits. We analyzed the categorical variables in the data set using a two-sample z-test to determine whether the two proportions of visits in each semester (Spring, 2019 and Fall, 2019) are equal. Two-sample Z-test is a generalization for the two-sample t-test when the sample is large, as is the case in this paper. Verma et al. (2019) mentioned that the two-sample t-test is an excellent method to measure the difference in proportions between two subsamples. Pearson Chi-square test for independence is used to assess the association between the independent variable (i.e., help-seeking behavior) and the dependent variable (i.e., success and persistence). According to Franke et al. (2012) and Mchugh (2013), Pearson’s chi-square tests are one of the most used tests that measure the association and difference between two or more categories.

The Chi-square test is a non-parametric test. It does not assume that the data follow a specific distribution, which is one of its strengths. However, the chi-square test requires that the sample size be more than 30, which is the case in this paper. The Chi-square test of association ($\chi^{2}$) is used because the help-seeking behavior, success, and persistence are categorical (nominal and binary) variables. Agresti (2018) stated that the larger the value of $\chi^{2}$ with a level of significance (p<.05), more evidence exists toward association (i.e., against the null hypothesis $H_{{}^{\circ}}:independence$). Then, a Chi-Square test for homogeneity is used on every single demographic variable that can be split into two or more different groups. For example, gender can be divided into females and males. In this study, each student's demographic variable entered the homogeneity test to determine whether help-seeking behavior differed significantly among each group. A significance value of .05 indicates significance in the homogeneity analysis. Assumptions of the homogeneity test, (a) the demographic variable groups are sampled randomly and (b) the demographic variable is categorical, are checked before running the test.

## 3 Results from research

### 3.1 Sample and service utilization description

The service utilization study was motivated by the institution’s desire to understand students’ help-seeking behavior as indicated through their use of support services. The study also served as a measure to close the loop for at-risk interventions carried out by the academic advising services on campus-to gauge how many of the students who were referred to the various campus and university support services pro-actively availed of the services. The sample for the present investigation included a total of *N* = 15,650 and *N* = 14,615 undergraduate students for Spring 2019 and Fall 2019, respectively. It is worth mentioning that 1,487 students and 3,778 students were omitted from the dataset because they were newly admitted in either semester and did not possess a GPA score.

The support services are categorized into two types: Institutional Support Services and College Support Services, as evidenced by Table 1 above. The service utilization for the Success Oases in various colleges includes the active registered student population eligible to use services or course support in each individual college. In Spring (2019), 10,174 visits were recorded to AAC (65.0%), which also has the most frequent visits compared to other support providers. In the context of colleges’ Success Oasis, it is noteworthy that no data was provided for the investigation of CED Success Oasis because service utilization was not tracked during Spring 2019. About 22.0% of all eligible students to avail of the services provided by the CAS Success Oasis, based on the courses offered, utilized the services in Spring (2019), 15.7% of students used the Foundation support services, and 27.3% visited the CENG success oasis. However, only 3.6% of the eligible students visited the LAWC Success Oasis to avail help. In the Fall of 2019, a large majority (66.4%) visited AAC, while 18.9% visited SLSC. Percentages in Table 2 represent the utilization of other services in Fall (2019).

**Table 2:** Campus service providers with corresponding student visits.

Service Provider	Semester			
	Spring 2019		Fall 2019	
	Overall	Student Visits	Overall	Student Visits
Institutional Support Service				
SLSC	*n* = 15,650	2,463 (15.7%)	*n* = 14,615	2,766 (18.9%)
CDC	*n* = 15,650	1,958 (12.5%)	*n* = 14,615	741 (5.1%)
AAC	*n* = 15,650	10,174 (65.0%)	*n* = 14,615	9,708 (66.4%)
College Success Oasis				
CAS	*n* = 3,666	805 (22.0%)	*n* = 3,442	293 (8.5%)
CBE	*n* = 4,245	548 (12.9%)	*n* = 3,869	1,088 (28.1%)
CENG	*n* = 1,310	357 (27.3%)	*n* = 1,090	233 (21.4%)
CSIS	*n* = 105	11 (10.5%)	*n* = 559	27 (4.8%)
CEDU	*n* = 0	-	*n* = 1,563	84 (5.4%)
LAWC	*n* = 1,475	53 (3.6%)	*n* = 1,523	274 (18.0%)
FN	*n* = 5,081	797 (15.7%)	*n* = 4,198	508 (12.1%)

The two-proportion Z-test was used to investigate whether the proportion of visits in the two semesters is comparable at a 5% significance level. The analysis confirmed a difference in the service utilization proportions in the two semesters for all the support service centers. In other words, there is a significant difference in students' behavior toward using support centers in Spring (2019) and Fall 2019.

**Figure 1:**
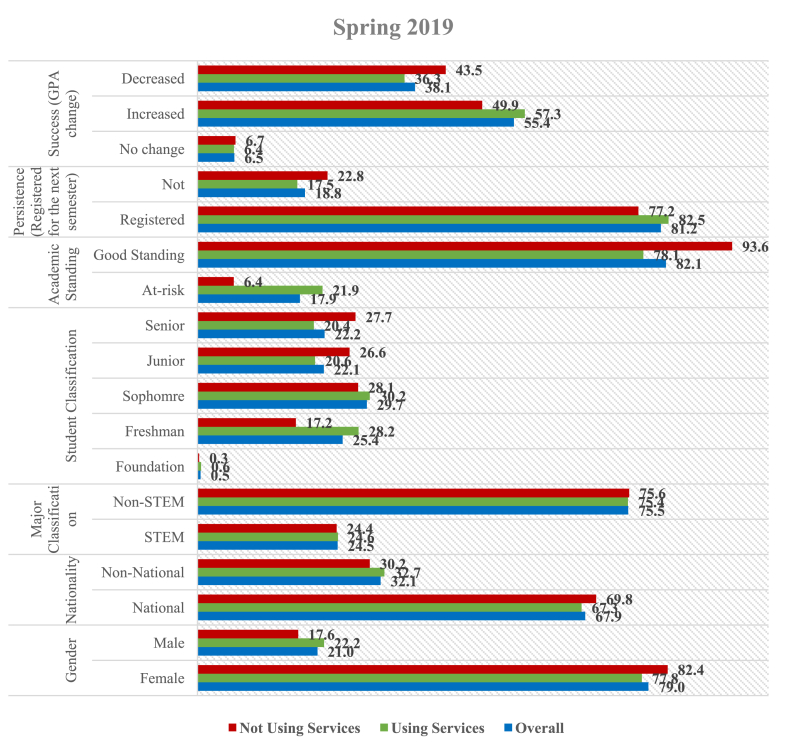
Student characteristics (Spring 2019) – in percentages.

**Figure 2:**
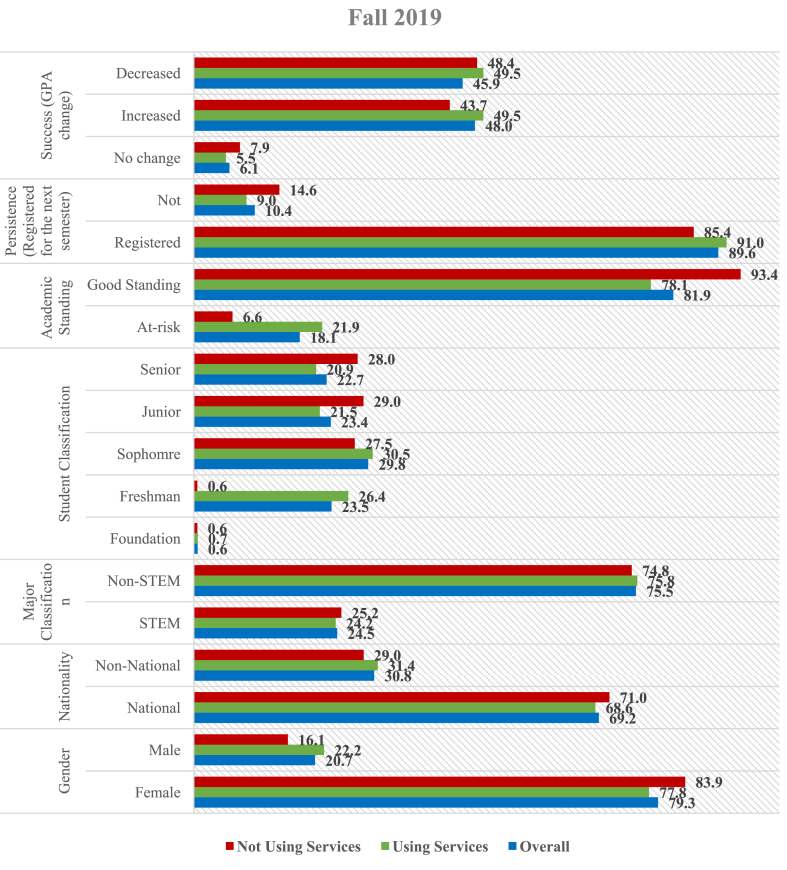
Student characteristics (Fall 2019) – in percentages.

Figures 1 and 2 below compare the students' help-seeking behavior across the Institutional Support Service centers and the Success Oases within colleges for the overall sample and sub-sample of at-risk students. In Spring (2019), the use of AAC among the general sample (65.0%) and at-risk students (88.5%) was significantly higher than in other support service centers. Figure 2 reports similar findings during the Fall of 2019. However, student utilization of AAC services was slightly lower in Spring (2019) (65.0%) than in Fall (2019) (66.4%). In Spring (2019), findings indicated that the proportion of at-risk students who used CDC services once (9.9%) was considerably higher than those who used these services twice or more (0.4%). Most frequently, in Spring (2019), at-risk students used the services of SLSC twice or more (7.5%). Besides, a slightly higher proportion of at-risk students used SLSC services once (9.5%) in the Fall of 2019 compared to those who used SLSC services twice or more (7.7%).

Among the colleges’ Success Oasis, data was unavailable for the CEDU Success Oasis in Spring (2019), and the sample population in CSIS indicated the lowest compared with other colleges. For instance, only 105 students are included in the study sample for Spring 2019, where 25 are recognized as at-risk students. CENG recorded the highest student utilization in Spring (2019) (27.3%), while in Fall (2019), CBE had the highest record (28.1%). Similar findings are observed for at-risk students' utilization of CENG (21.1%) and CBE (29.6%). About 10.6% of the overall students used CAS Success Oasis services once in the Spring of 2019, and 11.4% used the services twice or more. A decline was observed in the usage of services in CAS, wherein in the Fall of 2019, only 4.9% used CAS support services once, and 3.6% used these services twice or more. The lowest service utilization among the at-risk students was observed in the services of LAWC Success Oasis in Spring (2019) (2.1%) and CAS Success Oasis during Fall 2019 (4.4%).

Overall, 35.0% of the sampled students reported not using the AAC in the Spring of 2019. While among the at-risk students, only 11.6% did not use the support services of AAC. However, in the Fall of 2019, the numbers were very close, where 33.6% of the sampled students did not use AAC compared to 11.3% of the at-risk students. On average, 72.6% of the overall sampled students in Spring (2019) have not utilized any support services offered by the six colleges' Success Oasis and Foundation Program. This average increased in the Fall of 2019 to reach 86.0%. Statistics for at-risk students indicated comparable results to the overall sampled students in both semesters.

In all, 11,666 is the total number of students who visited at least one support service center in Spring (2019). Similarly, 10,960 students were reported in the Fall of 2019. Most students who utilized the support services for the two semesters are females. Most students who gained support from the campus resources are nationals (67.9% in Spring, 2019 and 69.2% in Fall, 2019), and only 32.7% of non-national students reached these support centers. Students in Non-STEM disciplines outnumbered their STEM counterparts in the usage of the support services within the two semesters. Only 17.9% (18.1 %) of the students who got help at any of the support service centers in Spring (2019) (Fall, 2019) were at risk.

**Figure 3:**
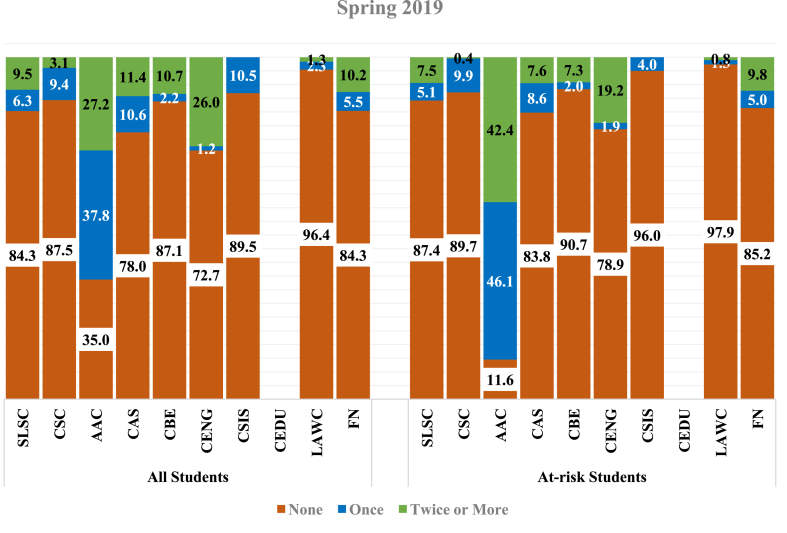
Usage of support services (Spring 2019) – in percentages.

**Figure 4:**
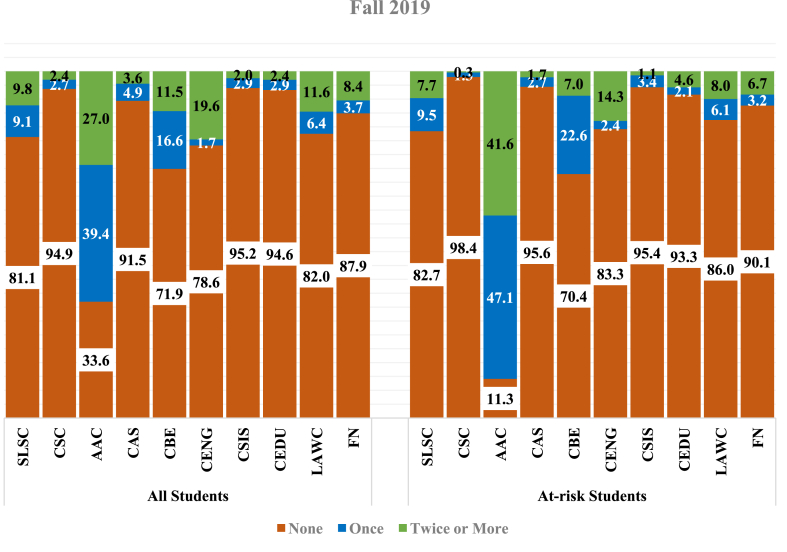
Usage of support services (Fall 2019) – in percentages.

Since this study focuses on the service utilization by QU students, Figures 3 and 4 provide parallel descriptive statistics between a sub-sample of students who used campus support services and another subsample of those who did not, for Spring 2019 and Fall 2019, respectively. This allows examination of the differences between the characteristics of help-seeking students and their counterparts. In most instances, students who seek help from the campus service centers are at-risk. As illustrated in Figure 3, the proportion of at-risk students is higher among those using services than in the overall sample. As to student classification, the number of students in the foundation who used services and those who did not are similar to the general sample. The number of first year (28.2%) and sophomore (30.2%) students are found to be over-represented among those who used services compared to those who did not overall. Correspondingly, juniors and seniors tend to be under-represented among those using services. Following the research aim, the number of students in Spring (2019) who used the campus support services represented a higher percentage of success (57.3% increase in GPA) and persistence (82.5% registered for next semester) in comparison with the overall sample and those who did not use any service.

Likewise, the proportion of female students in Fall (2019) who used services is lower than that in the other two categories, while the proportion of male students who used the services is more than in the other categories, as shown in Figure 4. This means that the proportion of females in the sample who used the support services is less than the proportion of males included in the sample who used the services. About 50% of the students who used the support services improved their GPA at the end of Fall (2019), and 91% registered for the next semester. This indicates a higher percentage of success and persistence among those using services than their counterparts.

### 3.2 Association measurements

As part of the analysis, it is interesting to determine the independent relationship between the students' help-seeking behavior and their success. This study will examine the relationships between the use of at-risk student services and student persistence and retention, based on factors such as gender, nationality, major, and student classification. This will enable universities to more effectively target their student support services to meet students' specific needs. The hypothesis to be tested:

H_0_: There is no association between the students’ help — seeking behavior and their success.
H_a_: There is an association between the students’ help — seeking behavior and their success.

A chi-squared test is undertaken for the data in Spring (2019) (see Table 3), and the results ($\chi^{2}=70.4,df=2,p<.05$) show that the null hypothesis is to be rejected, which means that there is an association between the two variables, in other words, students who used the services in Spring (2019) were more successful. A similar test was made for the data in Fall (2019) ($\chi^{2}=69.3,df=2,p<.05)$. The results indicate that the null hypothesis is rejected, which means there is a relation between the usage of the services and students’ success.

**Table 3:** Association measure between service utilization and success and persistence in Spring (2019) and Fall 2019.

		Spring 2019		Fall 2019	
		Overall	At-risk	Overall	At-risk
Success					
	Pearson Chi-square	70.45	69.34	51.65	60.13
	df	2	2	2	2
	P value	0.00	0.00	0.00	0.00
	N	15,650	2,809	14,615	2,643
Persistence					
	Pearson Chi-square	54.15	40.189	93.05	72.58
	df	2	2	2	2
	P value	0.00	0.00	0.00	0.00
	N	15,650	2,809	14,615	2,643

Regarding the association between the utilization of campus support services and students' persistence among the overall sample in Spring (2019), Table 4 presents the cross-tabulation for the data linking the use of campus support services and the registration for the next semester during Spring 2019. Pearson Chi-square statistic ($\chi^{2}=54.2,df=2,p<.05)$ indicated a statistical association between the usage of support services and students’ persistence. One can also infer that given that students used the support services provided by the university, 82.5% of them will persist by registering for the next semester, as in Table 4.

**Table 4:** Usage of Services∗Registered for next semester cross-tabulation (all students in Spring, 2019).

		Registered for next semester			
		Registered		Not	
		N	%	N	%
Usage of services	Used	9,626	**82.5**	2,040	17.5
	Not Used	3,077	77.2	907	22.8

On the other hand, Pearson Chi-square statistic ($\chi^{2}=40.2,df=2,p<.05)$ indicated a correlation between the usage of support services and the persistence of the at-risk students in Spring (2019). From Table 5, 77.2% of the at-risk students who used the support services in Spring (2019) will register for the next semester. Similar results were obtained in the Fall of 2019. However, the values of Chi-square are nearly double, providing more substantial evidence that a high association exists.

**Table 5:** Usage of Services∗Registered for next semester cross-tabulation (at-risk students in Spring, 2019).

		Registered for next semester			
		Registered		Not	
		N	%	n	%
Usage of services	Used	1,974	**77.2**	582	22.8
	Not Used	150	59.3	103	40.7

### 3.3 The difference in help-seeking behavior

This research also investigated whether the help-seeking behavior differs among the categories of students' characteristic variables. The Chi-square test for homogeneity is conducted to determine the difference between the proportions of the two categories of gender, nationality, major classification, and academic standing on help-seeking behavior. The null and alternative hypotheses are stated as.

H0: The Proportions of the two groups are equal.
Ha: The Proportions of the two groups are different.

Some assumptions on the variable’s nature are required to run the correct Chi-square homogeneity test. Hence, help-seeking behavior is one dependent variable that identifies whether the student has visited any support service center at least once. It is measured at a dichotomous level, including two categories: “used services” and “did not use services”, however, since our interest is the in the behavior of students who used the services, the proportions used are for the students who acquired a specific characteristic and used the support service centers. Each of the former students' characteristics is an independent variable consisting of only two categories, whereas students’ classification includes five categories.

The Null Hypotheses to be tested are:

H_0_: The proportions of males and females are the same
H_0_: The proportions of nationals and non — nationals are the same,
H_0_: The proportions of STEM and non — STEM are the same
H_0_: The proportions of foundation, first year, sophomore, junior, and senior are the same

In Spring (2019), a statistically significant difference in proportions of 0.051 in the help-seeking behavior between males and females was found. Help-seeking behavior for males (78.6%) is statistically higher than for females (73.5%). Likewise, the null hypothesis is rejected for nationality because the help-seeking behavior of non-nationals (76.1%) is higher than nationals (73.8%), with a significance value of *p* = .003. For students’ majors, we failed to reject the null hypothesis for the primary classification, and the binomial proportions are not statistically different, with a significance value of *p* = .764. Students in STEM majors tend to have similar help-seeking behavior (74.7%) as non-STEM students (74.5%). In addition, students classified as foundation (85.9%) and first-year (82.7%) have higher help-seeking behavior than their counterparts. Hence, the Chi-square test for homogeneity indicated sufficient evidence of a significant difference between three of the five student classification categories.

A parallel analysis was conducted only for the students whose academic standing is identified as at-risk. Unlike the overall sample, the help-seeking behavior is not statistically different for the gender of students at risk, with a significance value of *p* = .07. Furthermore, the proportion of help-seeking behavior is comparable among the categories of at-risk students’ nationality (*p* = .795) and primary classification (*p* = .058) (see Table 6).

**Table 6:** Proportion of services usage among students’ demographics (overall sample).

	Gender		Nationality		Major		Student Classification				
	Female	Male	National	Non-National	STEM	Non-STEM	Foundation	First-year	Sophomore	Junior	Senior
Spring 2019											
Usage of Services	9080 (73.5%)	2586 (78.6%)	7848 (73.8%)	3818 (76.1%)	2871 (74.7%)	8795 (74.5%)	73 (85.9%)	3291 (82.7%)	3521 (75.9%)	2402 (69.4%)	2379 (68.3%)
Fall 2019											
Usage of Services	8522 (73.5%)	2438 (80.6%)	7517 (74.3%)	3443 (76.5%)	2657 (74.3%)	8303 (75.2%)	72 (76.6%)	2891 (84.1%)	3348 (76.9%)	2358 (69.0%)	2291 (69.1%)

For both demographic variables. However, the Chi-square test for the help-seeking behavior among the five categories of at-risk student classification indicates a similar conclusion as the overall sample, with a significance value of *p* < .0001. Table 7 illustrates the proportions of at-risk students who used the support service centers concerning the different categories of students' demographic variables. For example, it can be said that 91.6% of the at-risk female students used support services in the Spring of 2019.

**Table 7:** Proportion of services usage among students’ demographics (at-risk students).

	Gender		Nationality		Major		Student Classification				
	Female	Male	National	Non-National	STEM	Non-STEM	Foundation	First-year	Sophomore	Junior	Senior
Spring 2019											
Usage of Services	1921 (91.6%)	635 (89.3%)	2088 (91.1%)	468 (90.7%)	610 (92.8%)	1946 (90.4%)	15 (75.0%)	890 (87.9%)	902 (93.3%)	458 (94.4%)	291 (89.5%)
Fall 2019											
Usage of Services	1817 (91.1%)	585 (90.1%)	1961 (90.5%)	441 (92.8%)	553 (93.1%)	1849 (90.2%)	20 (83.3%)	864 (90.9%)	806 (93.2%)	406 (89.2%)	306 (87.7%)

The Chi-square test in Fall (2019) indicates comparable results to the previous semester (Spring, 2019) among the help-seeking behavior for all of the listed demographic variables. However, the test of two proportions reveals a significant difference in proportions of 0.029 for help-seeking behavior between STEM and non-STEM students whose academic standing is classified as at-risk, *p* = .033.

## 4 Discussion

This study examined how campus support services substantially influence students' help-seeking behavior. The results indicate that students are encouraged to use support services offered by success oases and institutional support centers (questions 1a & 1b). According to Julal (2013), students are likely to stay in an educational institution with various support services available. Still, how individuals handle stress can avert some students from seeking help. Academics can use research that recognizes students who probably seek and use student support services so professors can help during their studies. Research can also be used to identify ways to improve access to and increase the use of support services. In this regard, these results indicate that in Spring (2019), AAC had the most frequent visits, and LAWC had the lowest. While in the Fall of 2019, AAC had the most visits, CSIS had the least. Zolezzi et al. (2017) explain that stigma is considered a common dilemma in mental health that creates hurdles for students in Qatar based on their gender and college type. More specifically, they found that stigmatizing attitudes held by most students included the belief that “people with mental illness cannot have regular jobs” (p. 1221). Therefore, it could be considered that moving toward institutional support services such as AAC is an outcome of a student's constructive behavior in seeking help.

Additionally, this study has found a significant association between service utilization and student success and persistence (questions 2a, b, and 3a, b). Students in general and at-risk students who utilized the support services were more likely to succeed and persist in their academic journey by returning to register for their next semester. In this regard, several prior research studies have confirmed that when students seek help from institutional services, it encourages them to be satisfied and exhibit higher performance (Brown et al., 2020; Khoury, 2017; Seeto, 2016). Furthermore, Khoury (2017) has confirmed that colleges with student support services enable students to avail themselves of the opportunity to perform better academically. Similarly, Seeto (2016) also stated that universities' effective student support services predict persistence in students' academic performance. When students face challenges and adopt help-seeking behavior from an institutional source, they tend to demonstrate higher performance in the long run due to consistent mentoring by university programs (Beisler and Medaille, 2016). The possible risks include lower awareness of academic courses, university projects, personal issues, and experiencing lower mental health to learn modules (Brown et al., 2020). Such problems can be tackled by an effective student support services program in a university. Therefore, it can be considered that using services in help-seeking behavior while experiencing risk predicts students' success and persistence.

In both semesters, there is a difference in the help-seeking behavior among the overall students, males and females, nationals and non-nationals, and student classifications (questions 4a & 4b). In contrast, students in STEM and non-STEM majors show similar help-seeking behavior. These findings are identical to several prior research studies (Al-Darmaki, 2011; Arthur, 2017; Rafal et al., 2018). Rafal et al. (2018) claimed that males expressed a higher need to demonstrate help-seeking compared to females. Arthur (2017) stated that international students are encouraged to seek help from institutional sources because of engagement, culture, and learning differences. Gansemer-Topf et al. (2017) claimed that students with STEM majors demonstrate higher help-seeking behavior than non-STEM students. This may imply that students require an appropriate level of awareness, then such demographic variables have a lower tendency to influence their help-seeking behavior (Sontag-Padilla et al., 2018). From this perspective, it can be considered that students with help-seeking behavior, based on these demographic variables, have higher student support services awareness than others.

Furthermore, results have indicated a significant difference between the at-risk student classification categories in Spring (2019). A difference in the help-seeking behavior between the at-risk student classification categories and the at-risk students in STEM and non-STEM majors in the Fall of 2019 was also found. In comparison, there is no difference in the help-seeking behavior among the demographic variables of the at-risk students other than the ones mentioned above. Gansemer-Topf et al. (2017) also claimed significant differences in students' persistence based on their affiliation with STEM and non-STEM. It is possible because students with STEM majors experience constructive issues that require complete support services (Sellami et al., 2017; Sithole et al., 2017).

Similarly, this study did not find any differences in gender and nationality toward help-seeking behavior in the two semesters. Students with sufficient awareness of support services can demonstrate help-seeking behavior regardless of the time (Sontag-Padilla et al., 2018). From this perspective, it can be considered that students demonstrated help-seeking behavior based on their awareness of the student’s support services.

## 5 Conclusion

Our key findings from this study indicated that students were encouraged to use support services. In both semesters, the Academic Advising Center had the most frequent visits. In the spring, the College of Law had the lowest number of visits, while in the fall, the College of Sharia and Islamic Studies was the lowest number of visits. Additionally, there was a significant association between service utilization and student success and persistence, students in general and at-risk students who utilized the support services were more likely to succeed and persist in their academic journey by returning to register for their next semester. Furthermore, Differences appeared between students, males and females, nationals and non-nationals, and student classifications in self-seeking behavior. At the same time, STEM and non-STEM majors show similar help-seeking behavior. There is no difference in the help-seeking behavior among the demographic variables of the at-risk students other than the ones mentioned above. Finally, there were no differences in gender and nationality toward help-seeking in the two semesters.

### 5.1 Limitations and further research

The goal of on-campus service delivery is to ensure students have the proper support for the right problem at the right time, so they can successfully progress in their academic journey. Thus, the findings in the current study have the potential to have implications for policymakers, service planners, and strategic planning of institutional resources and budgets.

Future research should focus on expanding efforts to explore service utilization at different types of universities in Qatar, as the services offered to vary significantly by the institution and needs of the student population it hosts. There is a deficit in research pertaining to student services and affairs in the MENA region, and similar studies published would help understand the type of services that are more utilized and efficient among students in the Arab world. This could guide institutions to benchmark their efforts and ‘tailor-make’ their services to cater to their student population's unique needs and demographics.

In addition, students should investigate the issues surrounding why they do not seek services and ensure that preventable barriers, such as stigma about seeking help, lack of necessary services, and laissez-faire policies, are not the cause.

Finally, additional studies could examine and research the service utilization among pocket populations in university campuses among non-national students, international students, students with special needs, working students, non-traditional mature students, etc. Several studies have considered these aspects in a broad sense (Kerr et al., 2013; Miranda et al., 2015). Few studies examined the relationship between the specific issues and services utilized. For example, Miranda et al. (2015) reported that racial minorities reported having more hurdles and challenges accessing on-campus counseling services for mental health issues than white students. Combining this knowledge with current research results, one of the future directions is to look at demographic characteristics as an intermediary in the relationship between students' risky academic status and on-campus service use. Such information may provide strong evidence for more comprehensive coordination of on-campus services and awareness.

In this study, data collection was challenging, as it required continuous tracking of the service utilization by students manually from multiple support providers throughout the semester. The research process could inform policymakers of the need to automate monitoring of student service utilization and inform them of services most in need of such tracking to plan their projects. Due to privacy concerns, essential student support services were not considered in the study (e.g., student counseling and unique need support services). Future efforts need to include these student populations without violating student confidentiality. The current research focuses mainly on undergraduate students. Future work may consider service utilization for graduate students. The data collected that was available with support providers were basic; hence, for this study, minimal statistical analysis techniques were deployed. A future study could expand on this by obtaining more information about the students to deploy expanded analytical techniques. Despite gaps in knowledge about using services on campus, current results can help with planning at similar universities. Our results suggest that certain services have higher student usage than others, and it is worth exploring the factors that mitigate this trend.

## Declarations

### Author contribution statement

Chithira Johnson; Abdel-Salam G. Abdel-Salam; Radwa Ismail: Conceived and designed the experiments; Analyzed and interpreted the data; Wrote the paper.

Rizwan Gitay; Rusol Adil Naji Al-Tameemi: Performed the experiments; Analyzed and interpreted the data.

Ahmed BenSaid: Analyzed and interpreted the data.

Michael H. Romanowski; Batoul Mohamad Kazem Al Fakih: Analyzed and interpreted the data; Wrote the paper.

Khalifa A. Haza: Conceived and designed the experiments; Wrote the paper.

### Funding statement

This research did not receive any specific grant from funding agencies in the public, commercial, or not-for-profit sectors.

### Data availability statement

The authors do not have permission to share data.

### Declaration of interest’s statement

The authors declare no competing interests.

### Additional information

No additional information is available for this paper.

## Figures and Tables

**Table:** Major Classifications

Qatar University Major	Classification		
Architecture	STEM		
Biology	STEM		
Biomedical Sciences	STEM		
Chemical Engineering	STEM		
Chemistry	STEM		
Civil Engineering	STEM		
Computer Engineering	STEM		
Computer Science	STEM		
Electrical Engineering	STEM		
Environmental Sciences	STEM		
Human Nutrition	STEM		
Industrial and System Eng.	STEM		
Mathematics	STEM		
Mechanical Engineering	STEM		
Medicine	STEM		
Pharmacy	STEM		
Public Health	STEM		
Sport Science	STEM		
Statistics	STEM		
Dental Medicine	STEM		
Accounting	Non STEM		
Arabic Language	Non STEM		
Creed and Dawa	Non STEM		
Dawa	Non STEM		
Economics	Non STEM		
English Literature & Linguistics	Non STEM		
Finance	Non STEM		
Fiqh and Usul Al Fiqh	Non STEM		
History	Non STEM		
International Affairs	Non STEM		
Islamic Studies	Non STEM		
Law	Non STEM		
Management	Non STEM		
Management Information System	Non STEM		
Marketing	Non STEM		
Mass Communication	Non STEM		
Physical Education	Non STEM		
Policy, Planning, and Dev	Non STEM		
Psychology	Non STEM		
Quran and Sunnah	Non STEM		
Social Work	Non STEM		
Sociology	Non STEM		
Special Education	Non STEM		
Primary Education	*STEM/Non-STEM depends on students concentration*	Arabic Studies, Early Childhood, English Language Non-STEM	Mathematics & Sciences STEM
Secondary Education	*STEM/Non-STEM depends on students concentration*	Arabic Language, English, Islamic Studies, Social Sciences Non-STEM	Biology, Chemistry, MathematicsSTEM
